# Technical note: Temperature estimation accuracy based on colourimetry of embalmed human and fresh non-human burned bone

**DOI:** 10.1007/s00414-024-03239-7

**Published:** 2024-04-26

**Authors:** Tristan Krap, Afke Leenstra, Roelof-Jan Oostra, Wilma Duijst

**Affiliations:** 1 Faculty of Law and Criminology, Maastricht University, Minderbroedersweg 4-6, Maastricht, 6211 LK The Netherlands; 2 Forensic Laboratory Research, University of Applied Sciences Van Hall Larenstein, Agora 1, Leeuwarden, 8934 CJ The Netherlands; 3 Department of Medical Biology, Section Clinical Anatomy and Embryology, Location Academic Medical Centre, Amsterdam Medical Centre, Meibergdreef 9, Amsterdam, 1105 AZ The Netherlands

**Keywords:** Forensic anthropology, Fire, Cremation, Heated bone

## Abstract

Estimation of the exposure temperature of skeletal remains can be done by means of colourimetry and a previously published decision model for the colourimetric data, resulting in clusters that represent a range of exposure temperature. The method was based on thermally altered freshly burned human skeletal remains. However, in practice the origin or pre-burning condition of (possibly fragmentary) burned remains can be unknown. Further, in order to use the colourimetric analysis, and accompanying decision model, it is important to have a reference or test set. Fresh human material is not available for this purpose in all countries. Hence, the classification accuracy of the decision model was tested for, in some countries more readily available substitutes for fresh bone; embalmed human bone and non-human bone. The model yielded high accuracies for these sample materials, making it possible to create a reference or test set from fresh non-human and embalmed human bone as substitute for human, and also to use the decision model for these deviating samples in practice.

**Supplementary Information:**

The online version contains supplementary material available at 10.1007/s00414-024-03239-7.

## Introduction

Bone that is exposed to heat undergoes heat-induced (HI) molecular changes, and, as a result, discoloration occurs [1–5]. The major changes in bone colour are from either:

- carbonization of organic components, a process that occurs under both oxidizing and reducing conditions and starts from approximately 250 °C and upwards, and results grossly in a brown to black discoloration,
- combustion of carbon containing molecular debris embedded in the bone matrix, a process that only occurs under oxidizing conditions and sets in from approximately 450 °C and upwards, and results in shades of gray with different colour casts until combustion completes and white calcined bones remain [6–9].

Traditionally, these changes were assessed visually and by comparison with references charts, for example a heated bone discoloration chart or values of a Munsell colour atlas [1–3, 10]. These subjective approaches proved to be inaccurate, imprecise and did not uphold to the legal standards of evidence used in court [11, 12]. Therefore, objective methods were drafted that involve the use of measured colourimetric data, obtained from a calibrated imaging device [4, 13, 14]. One of the previously published methods makes use of the CieLAB colourspace, which is composed of a value for Lightness combined with two colour coordinates A and B (L*A*B*). The L and B value proved useful for clustering the HI colour change, with which a model for temperature exposure estimation was developed [4]. While precision differed per cluster, the model yielded an acceptable accuracy of, on average, 90%, based on either a calibrated DSLR camera or a flatbed scanner. The benefit of this method is the ease of application for practice, as the required devices are relatively inexpensive and in most cases readily available, it requires no specialized software (ImageJ is a free online tool), and the collection of colourimetric data from an image is a relative easy process.

For forensic practice it is important to validate methods, especially for laboratory that are ISO accredited (i.e. ISO/IEC 17,025 accreditation) [15]. In order to validate a method, a test set is needed. The possibility of using human remains, for scientific validation and calibration, differs per country. Fresh human skeletal material may be unobtainable or not legally available for this purpose. Therefore, it is necessary to test whether colourimetry can be validated by means of substitute materials, like embalmed human bone. 1 In embalmed material there is an increase in collagen fiber-cross connections making the structure more dense and rigid and therefore less prone to degradation [16].

It can, initially, be difficult, if not impossible, in practice to differentiate between fragments from human and non-human remains in fire case with severe fragmentation and without the aid of microscopy and analytical techniques [17, 18]. Therefore, it is relevant to know whether the colourimetric model for temperature estimation yields accurate results when used to estimate the exposure temperature of burned non-human bone fragments as well.

In this study we tested the classification accuracy of the previously proposed colourimetric method based on the CieLAB colourspace on thermally altered embalmed human bone samples and thermally altered non-embalmed non-human bone as a proxy for human.

## Materials and methodology

### Sample preparation and heating experiment

Human long bones: radii, ulnae, and humeri, were extracted from embalmed cadavers initially intended for (bio)medical dissection courses at the Amsterdam UMC, location AMC. In total 6 individuals contributed to the collection, of which 2 males with an age of 56 and 70 year and 4 females with an age range of 74 to 92 year. Non-human long bones: radii, ulnae, and humeri, from *Sus scrofa domesticus (dom.)* and long bones: radii, ulnae, tibia, and fibulae, from *Bos taurus*, were extracted from limbs classed as offal provided by a biological butchery. The diaphyseal part, of the acquired long bones, was manually defleshed, including removal of the periosteum, with the backside of the blade of a scalpel. The diaphysis were then sawn into transverse sections of approximately 4 mm thickness, in a layer of water (room temperature) to prevent heating of the sample by friction and aeration of bone dust. Bone samples were thereafter kept refrigerated between 3 °C and 6 °C.

The transverse sections were placed in porcelain cups and heated in a preheated muffle oven under plain air, with an accuracy of ± 2 °C, for 10 to 50 min at a temperature in the range of 50 °C to 1000 °C with incremental steps of 50 °C to 100 °C. For this study, 293 human embalmed bone samples, 106 *Sus Scrofa dom*. bone samples, and 49 *Bos taurus* bone samples (total *N* = 448) were used for this study, divided over the temperature-duration groups, see the electronic supplement for an overview (ESM table s1). After heating, the samples were left to cool down to room temperature, placed in labeled plastic tissue cassettes and stored at room temperature in a dark and ventilated cabin.

### Data collection and statistical analysis

Samples were scanned with a flatbed scanner, Epson perfection v10, at 300 DPI with a white background. Calibration was carried out with a Spydercheckr 24. Images were exported as uncompressed files (TIFF). The colourspace of the images was then converted to L*A*B* in ImageJ supplemented with plug in Colour Transformer. The average L* and B* values were collected from the cortical surface of the transverse sections, for which the outer (periosteal layer) and inner rims (endosteal layer) of the bone sample were excluded from the colourimetric analysis due to overexposure and chromatic abbreviation.

The L and B values of the heated bone samples were then used to define the associated temperature estimation cluster based on previously published decision rules, see Table 1 [4]. The classification accuracy was calculated as follows. In case the actual exposure temperature of the sample fell within the range of the estimated exposure temperature the estimation was classed as correct. In case the actual exposure temperature fell outside of the exposure range the temperature estimation was classed as incorrect. The accuracy was then calculated as the percentage of correct estimations for each of the categories (non-human embalmed, *Sus Scrofa dom*. and *Bos taurus)*, rounded to one decimal place.

**Table 1:** Decision rules for the temperature estimation based on L* and B* colour values [4]

Cluster	Decision rule	Minimum temperature	Maximum temperature
1	L > 40 and B > 11	0 °C	350 °C
2	L < 40 and B > 11	250 °C	350 °C
3	L < 32,5 and B < 11	300 °C	600 °C
4	(32,5 < L < 75) and B < 11	450 °C	600 °C
5	(32,5 < L < 75) and B < 6,5	450 °C	700 °C
6	L > 75 en B < 11 and L < (-25*B + 200)	700 °C	> 900 °C
7	L > 75 en B < 11 and L > (-25*B + 200)	800 °C	> 900 °C

## Results

**Fig. 1:**
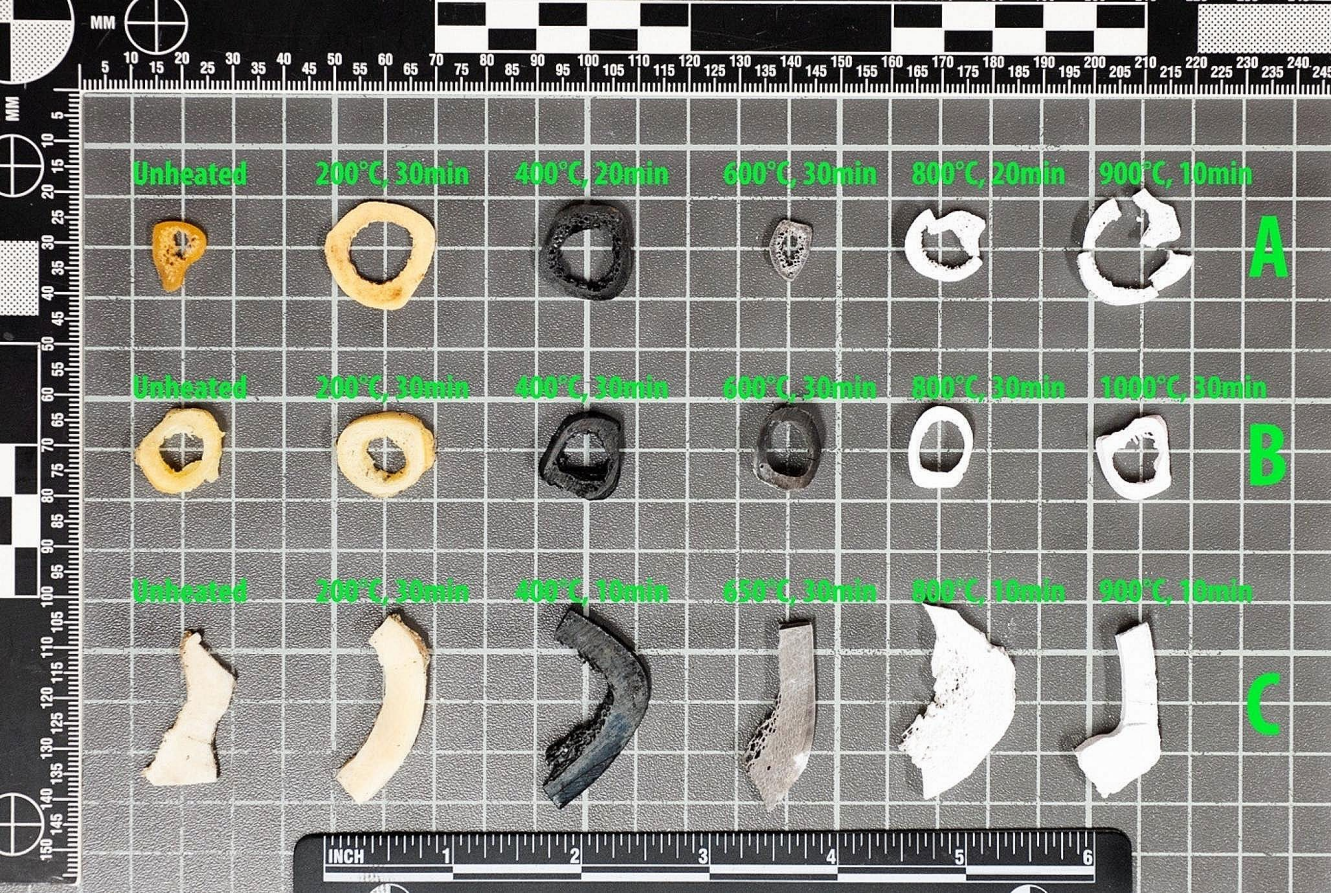
Selection of samples from the three groups of heated bone sections with exposure temperature (°C) and duration (minutes)*, **A:** Embalmed human, **B:** Pig (*Sus scrofa dom.*), **C:** Cow (*Bos taurus*). *This image is intended as overview and not suitable for colourimetric analysis

A selection of samples, taken from the three groups, is displayed in Fig. 1. The temperature of heated human embalmed bone samples was correctly estimated in 99.7% of the cases. Of the 293 samples, a single sample was overestimated (220 °C for 30 min was classed as 250 °C to 350 °C). See Table 2.

**Table 2:** Accuracies for the colourimetric model, applied to human (embalmed) and non-human skeletal material that was exposed to heat in the range of room temperature to 1000 °C *

Species	*N*	Accuracy
Human, embalmed	293	99.7%
*Pig (Sus scrofa dom.)*	106	94.3%
*Cow (Bos taurus)*	*49*	*100.0%*
Non-human	155	97.2%

*See ESM-1 table s1 for a complete overview of sample distribution over the temperature-duration groups

The overall classification accuracy for the non-human samples was 97.2%. Of the 106 samples from *Sus scrofa dom*. six were classed incorrectly. Four of these samples were overestimated (250 °C for 20 to 30 min was classed as 300 °C to 600 °C) while the remaining two were underestimated (650 °C for 10 min was classed as 300 °C to 600 °C), resulting in a classification accuracy of 94.3%. None of the non-human samples from *Bos taurus* were classed incorrectly, resulting in a classification accuracy of 100%. See Table 2.

## Discussion

Previously the colourimetric decision model based on the L* and B* value proved to be highly accurate for freshly burned human bone sections that were heated in media air or adipose tissue, and even for samples that deviated in exposure duration from the exposure duration used to develop the model. Also, the model proved to be highly accurate for samples that deviated in size from the sections used to develop the model [4]. Now the model has also been tested on embalmed human and non-human bone samples, yielding a high accuracy. Overall, this shows that the colourimetric decision model is robust and broader applicable than originally intended.

The majority of the incorrect temperature estimations were overestimations of the temperature, five of seven incorrect estimates out of 448 samples, of which four from pig. The temperature range of the incorrect estimations was from 220 °C to 250 °C, with just an overestimation of a single cluster, while the exposure temperature of the two underestimations (of also a single cluster) was 650 °C.

The increase in inter- and intrafibrillar cross-links, from the formalin fixation of the collagen in the bone matrix, and the addition of formalin (containing organic components), did not have a measurable effect on the classification accuracy based on HI-changes in colour. This shows that the results from a previous study, on the accuracy and precision of temperature estimations based on embalmed human bone samples, are not influenced by the addition of formalin [11]. However, it remains unclear whether, or to what degree, formalin fixation has an effect on the thermal stability.

Bone mineral density (BMD) might influence the rate of combustion of the organic components, a higher BMD could negatively affect oxygen availability necessary for combusting the organic remnants especially after carbonization. Porcine bone has the highest BMD of the, for this study, used species, based on mid-diaphyseal long bone measurements (femur, tibia, humerus, radius, of which the cow tibia showed a questionable low value but even by using the less questionable distal or proximal value the average BMD for cow remained lower compared to pig), while cow bone has the highest mean volumeBMD (449 mg/cm^3^) followed by pig (373 mg/cm^3^) and human bone (178 mg/cm^3^) [19, 20]. The carbonization of pig bone actually preceded cow and human bone (resulting in overestimation); hence the higher BMD of pig cortical bone did not negatively affect carbonization. The combustion of the organic component of pig bone, at 650 °C, lacked behind for two samples, indicating that BMD may indeed effect combustion. The effect of BMD on HI-changes should be further investigated in the future. Besides BMD, the ratio inorganic versus organic, collagen integrity, and porosity can possibly affect carbonization and combustion of the organic component of bone, interspecies differences of these independent variables can lead to incorrect temperature estimations.

The number of incorrect classifications does not substantiate the exclusion of using the model. However, the chosen species for this study do not reflect all possible species that can be encountered in a fire context. Remains of domestic animals can be expected to be encountered amongst the debris after a fire, also a wider variety of species can be expected to be found during archaeological excavations. It is, therefore, necessary to expand this classification accuracy test, and until then refrain from drawing conclusions on samples of species of which the classification accuracy is unknown. Further, one cannot discriminate between fresh human, embalmed human and non-human bone (*Sus scrofa dom*. and *Bos taurus)* with the colourimetric model in cases in which the exposure temperature is known.

Recently a scale, containing a bone colour to exposure temperature chart, was suggested as a tool for temperature estimations [21], which, when used visually, does not deviate from the previously described subjective methods. As previously mentioned, the subjective approach did not uphold to the legal standards [11, 12]. Taking colourimetric measurements is more laborious and time consuming than visually assessing and comparing the colour, with for example reference charts, but the data acquired from colourimetry is objective. We advise to adopt the scale to the colourimetric decision model and incorporate reference colours to test the imaging capturing device (instead of visual comparison).

## Conclusion

The colourimetric decision model created for estimating the temperature of heated fresh human bone samples proved highly accurate for two deviating sample sets, namely embalmed human and fresh non-human bone containing pig (*Sus scrofa dom*.) and cow (*Bos taurus)*. Based on the high accuracies a reference, or test set, based on the tested substitutes, human embalmed bone and fresh non-human bone, for fresh human bone are valid options.

### Electronic supplementary material

Below is the link to the electronic supplementary material.

Supplementary Material 1

## Footnotes

1 In rare cases in practice, bone fragments salvaged from fire debris can originate from anatomical specimens that were kept as props, which can be chemically treated, and are not necessarily of human origin. Uncertainty on the origin of skeletal remains in such contexts leads to the demand for knowledge on the validity of the applied method.
