# Supplementary material for: Technical note: Temperature estimation accuracy based on colourimetry of embalmed human and fresh non-human burned bone
Source: Int J Legal Med. 2024 Apr 26;138(5):2107–11. doi: 10.1007/s00414-024-03239-7 (PMC11306499; doi:10.1007/s00414-024-03239-7)
Supplement: Supplementary file 1 — Supplementary Material 1 [file 414_2024_3239_MOESM1_ESM.docx]

**Technical note: Temperature estimation accuracy based on colourimetry of embalmed human and fresh non-human burned bone**

Tristan Krap ^1,2,3^, Afke Leenstra ^2^, Roelof-Jan Oostra ^3^, Wilma Duijst ^1^

1: Faculty of Law and Criminology, Maastricht University, Minderbroedersweg 4-6, 6211 LK Maastricht, The Netherlands

2: Forensic Laboratory Research, University of Applied Sciences Van Hall Larenstein, Agora 1, 8934 CJ Leeuwarden, The Netherlands

3: Department of Medical Biology, Section Clinical Anatomy and Embryology, Amsterdam Medical Centre, Location Academic Medical Centre, Meibergdreef 9, 1105 AZ Amsterdam, The Netherlands

**Electronic Supplement Material (ESM)**

For an overview of 293 human embalmed bone samples, 106 *Sus Scrova dom*. bone samples, and 49 *Bos taurus* bone samples (total N= 448) divided over the temperature-duration groups, see table s1.

**Table s1. Sample size per temperature-duration group for each sample class 1/2.**

| **Temperature** | **Duration (min)** | **Human (N)** | **Sus scrofa (N)** | **Bos taurus (N)** |
| --- | --- | --- | --- | --- |
| Unheated | - | 3 | 1 | 1 |
| 50°C | 20 | - | 2 | - |
|  | 30 | - | 2 | 2 |
| 100°C | 10 | 10 | 2 | - |
|  | 20 | 10 | 2 | 2 |
|  | 30 | 10 | 2 | - |
| 150°C | 10 | 9 | 2 | 2 |
|  | 20 | 9 | 2 | - |
|  | 30 | 9 | - | - |
|  | 40 | 10 | - | - |
|  | 50 | 9 | - | - |
| 180°C | 10 | 9 | - | - |
|  | 20 | 9 | - | - |
|  | 30 | 9 | - | - |
| 200°C | 10 | 9 | 2 |  |
|  | 20 | 9 | 2 |  |
|  | 30 | 9 | 2 | 2 |
| 220°C | 10 | 9 | - | - |
|  | 20 | 9 | - | - |
|  | 30 | 9 | - | - |
| 250°C | 10 | 10 | 2 | 2 |
|  | 20 | 10 | 2 | 2 |
|  | 30 | 9 | 2 |  |

**Table s1. Sample size per temperature-duration group for each sample class 2/2.**

| **Temperature** | **Duration (min)** | **Human (N)** | **Sus scrofa (N)** | **Bos Taurus (N)** |
| --- | --- | --- | --- | --- |
| 300°C | 10 | 10 | 2 | 2 |
|  | 20 | 10 | 2 | 2 |
|  | 30 | 10 | 2 | - |
| 350°C | 10 | 10 | 2 | - |
|  | 20 | 9 | 2 | - |
|  | 30 | 9 | 2 | 2 |
| 400°C | 10 | 8 | - | 2 |
|  | 20 | 9 | 2 | - |
|  | 30 | - | 2 | - |
| 450°C | 10 | - | 2 | 2 |
|  | 20 | - | 2 | 2 |
|  | 30 | 3 | 2 | - |
| 500°C | 10 | 3 | 2 | - |
|  | 20 | 2 | 2 | - |
|  | 30 | 2 | 2 | 2 |
| 550°C | 10 | - | 2 | 2 |
|  | 20 | - | 2 | - |
|  | 30 | - | 2 | - |
| 600°C | 10 | 3 | 2 | 2 |
|  | 20 | 2 | 2 | 2 |
|  | 30 | 2 | 2 | - |
| 650°C | 10 | - | 2 | - |
|  | 20 | - | 2 | - |
|  | 30 | - | 2 | 2 |
| 700°C | 10 | 2 | 2 | - |
|  | 20 | 2 | 2 | 2 |
|  | 30 | 2 | 2 | - |
| 750°C | 10 | - | 2 | - |
|  | 20 | - | 2 | 2 |
|  | 30 | - | - | - |
| 800°C | 10 | 2 | 2 | 2 |
|  | 20 | 2 | 2 | - |
|  | 30 | - | 2 | - |
| 900°C | 10 | 3 | 2 | 2 |
|  | 20 | - | 2 | 2 |
|  | 30 | - | 2 | - |
| 1000°C | 10 | - | 1 | - |
|  | 20 | - | 2 | - |
|  | 30 | - | 2 | - |
| **Total** | | 293 | 106 | 49 |
